# Potential use of human hair shaft keratin peptide signatures to distinguish gender and ethnicity

**DOI:** 10.7717/peerj.8248

**Published:** 2020-01-30

**Authors:** Nurdiena Mohamed Nasir, Jumriah Hiji, Jaime Jacqueline Jayapalan, Onn Haji Hashim

**Affiliations:** 1 Department of Molecular Medicine, Faculty of Medicine, University of Malaya, Kuala Lumpur, Wilayah Persekutuan, Malaysia; 2 University of Malaya Center for Proteomics Research, Faculty of Medicine, University of Malaya, Kuala Lumpur, Wilayah Persekutuan, Malaysia

**Keywords:** Human hair shaft proteins, 2DE, Mass spectrometry, Biomarkers, Alkaline solubilisation method

## Abstract

**Background:**

Most human hairs collected at old crime scenes do not contain nuclear DNA and are therefore of less value for forensic investigations. In the present study, hair shaft proteins were extracted from 40 healthy subjects between the ages of 21 to 40 years and profiled using gel electrophoresis-based proteomics to determine if they can be used to distinguish gender and ethnicity.

**Methods:**

Extraction of the human hair shaft proteins was performed using a newly developed alkaline solubilisation method. The extracts were profiled by 2-dimensional electrophoresis and resolved protein spots were identified by mass spectrometry and queried against the human hair database. The study was then followed-up by immunoblotting of the identified hair shaft keratin of interest using commercially available antibodies.

**Results:**

Separation of the human hair shaft proteins by 2-dimensional electrophoresis generated improved and highly resolved profiles. Comparing the hair shaft protein profiles of 10 female with 10 male subjects and their identification by mass spectrometry and query of the human hair database showed significant altered abundance of truncated/processed type-II keratin peptides K81 (two spots), K83 (one spot) and K86 (three spots). The 2-dimensional electrophoresis profiling of 30 hair shaft samples taken from women of similar age range but from three distinctive ethnic subpopulations in Malaysia further showed significant altered abundance of one type-I and four type-II truncated/processed keratin peptides including K33b, K81, K83 and K86 (2 spots) between at least two of the ethnic groups. When a followed-up immunoblotting experiment was performed to detect the relative expression of the K86 peptides using commercialised antibodies, similar trends of expression were obtained. The present data, when taken together, demonstrated the potential use of keratin peptide signatures of the human hair shaft to distinguish gender and ethnicity although this needs to be further substantiated in a larger scale study.

## Introduction

The hair shaft is formed from epidermal keratinocytes undergoing a keratinization programme cell death termed cornification. This process involves degradation and destruction of the cell nucleus as well as the genetic material enclosed within it (Eckhart et al., 2013). As a result, nuclear DNA which may be used for genetic fingerprinting is usually not detected in the hair shaft (Bender & Schneider, 2006). However, the application of next generation sequencing for the analysis of mitochondrial DNA, short tandem repeats, and single nucleotide polymorphisms has also demonstrated a great promise for forensically challenging samples (Shih et al., 2018). On the other hand, the potential of distinguishing individual hair donors based on their hair shaft proteins has also been highlighted (Wu et al., 2017).

The complex interaction of these hair shaft proteins provides robust rigidity to the hair structure and makes it resistant to many environmental factors such as pollutants, weather, ultraviolet light or chemical treatments (Wolfram, 2003). Despite being well conserved, previous studies have shown that characterization of the hair shaft proteins can be challenging and difficult. This is mainly due to difficulties in solubilizing and extracting the proteins in solvents that are compatible with liquid chromatography or gel electrophoresis approaches (Sun et al., 2014; Kollipara & Zahedi, 2013; Han et al., 2007; Smith & Parry, 2007; Langbein & Schweizer, 2005). While many attempts to improve the yield of proteins extracted from the human hair shaft have been reported, the quality of published 2-dimensional gel electrophoresis profiles is still far from being a useful method for forensic investigations and the process is also time-consuming and not practical to be applied in a large scale study (Fujii, Takayama & Ito, 2013; Barthélemy et al., 2012; Thibaut et al., 2009; Plowman, 2007; Nakamura et al., 2002). However, an alkaline solubilisation method which is capable of extracting substantially higher percentage of hair shaft proteins within only 2 h was recently developed (Wong et al., 2016). With this improved method and higher yield of hair shaft proteins, we analysed the hair material using 2-dimensional electrophoresis gel-based proteomics as well as immunodetection methods and compared the profiles to determine if they can be used to distinguish gender and/or ethnicity.

## Methodology

### Subject recruitment

A total of 40 healthy individuals of different genders and ethnicities but with the same age range (21–40 years) were recruited in the study in accordance to the ethical clearance granted by the Medical Ethics Committee of University of Malaya Medical Centre (MEC ID. NO: 20158-1577) (Institutional Review Board), which adheres to the Declaration of Helsinki. Informed written consent was acquired from all subjects prior to collection of their head hair samples. At least three strands of hair were collected from each subject and stored at −20 °C. In addition to visual inspection, information regarding subject’s hair treatment habits and relevant past medical histories, if any, were collected using a questionnaire. Relevant phenotypic characteristics of the hair shaft including discoloration, whitening, bleaching, weathering and perming, if any, were recorded. Subjects with previous history of bacterial or fungal-borne skin diseases, inflammation or cancer and/or under treatment for such ailments and those with chemically-treated hair were excluded from the study.

### Isolation of human hair shaft proteins

Human hair shaft proteins were isolated using the alkaline solubilisation method in two stages as previously described (Wong et al., 2016; Budowle & Acton, 1981). Briefly, 5 mg of hair samples were sterilised with 90% ethanol, cut (1–4 mm) and incubated in 300 µl of lysis buffer at 90 °C for 30 min in the first stage. The resulting supernatant fluids were isolated using a QIAquick spin column (Qiagen, Hilden, Germany) and kept at 4 °C. The undissolved hair shaft fractions on the other hand, were transferred into a fresh tube containing 300 µl of lysis buffer with 0.1 M NaOH and pulverised by magnetic stirring for 30 min to increase the recovery of protein from the second stage of extraction. The resulting solutions containing hair shaft protein extract were centrifuged and the supernatant fluids were separated for subsequent precipitation of proteins using 4 °C acetone. Protein pellets were then solubilised in sample buffer and their concentrations were assayed using the Bio-Rad protein assay (Bio-Rad, Hercules, CA, USA) in accordance to the manufacturer’s guidelines.

### 2-dimensional electrophoresis and silver staining

Solubilised hair shaft proteins (100 µg) suspended in sample buffer were incubated in rehydration buffer containing 7 M urea, 2 M thiourea, 4% 3-[(3-cholamidopropyl) dimethylammonio]-1-propanesulfonate hydrate, 2% immobilized pH gradient buffer pH 4-7, 0.002% w/w orange G and 7 mg of dithiothreitol (DTT) for 30 min at room temperature. The sample mixtures were rehydrated with 13 cm DryStrip gel pH 4-7 (GE Healthcare, Uppsala, Sweden) for 18 h at room temperature in a closed environment. The rehydrated strips were subjected to first dimensional gel electrophoresis separation using the Ettan IPGphor 3 Isoelectric Focusing System (GE Healthcare, Uppsala, Sweden) according to the following settings: (i) 500 V, 1 h, step and hold; (ii) 1,000 V, 1 h, gradient; (iii) 8,000 V, 2 h 30 min, gradient; (iv) 8,000 V, 55 min, step and hold. The focused strip was initially incubated with SDS equilibration buffer consisting of 6 M urea, 75 mM Tris–HCl, pH 8.8, 30% (v/v) glycerol, 2% sodium dodecyl sulphate (SDS) and 1% (w/v) DTT for 20 min, followed by a second equilibration in the same buffer but containing 4.5% (w/v) iodoacetamide instead of DTT for another 20 min. Second dimension separation was performed on 8–15% gradient polyacrylamide gel using the SE 600 Ruby electrophoresis system (GE Healthcare, Uppsala, Sweden). A concentration of 2.6% crosslinker (Bis) was used for the preparation of gels (*% C*). Cathode running buffer containing 0.025 M tris (pH 8.3), 0.192 M glycine and 0.1% SDS while, the anode buffer containing 0.375 M tris (pH 8.8) were used for the second dimensional gel electrophoresis separation. Four gels were run according to the following program: (i) 50 V, 150 mA, 100 W for 1 hr; (ii) 600 V, 150 mA, 100 W until tracker dye reached bottom of the gel. The electrophoresed gels were stained using silver nitrate (Yan et al., 2000). In this process, gels were firstly fixed with 40% (v/v) ethanol and 10% acetic acid for 30 min, followed by sensitization with 40% (v/v) ethanol, 0.5 M sodium acetate trihydrate and 8 mM sodium thiosulphate for 30 min. Gels were then washed thrice, each time for 5 min, and stained with 14.7 mM silver nitrate solution. After washing two more times for 1 min, gels were developed with 0.24 M sodium carbonate and 0.04% (v/v) formaldehyde. Development of spots was stopped with 40 mM EDTA solution and gels were finally kept in distilled water before being scanned (see next section). Subsequent to the present study, a report on modification of this 2-dimensional electrophoresis method, which generated further improved image resolution, was published (Wong, Hashim & Hayashi, 2019).

### Data analysis

Silver-stained 2-dimensional electrophoresis gels were scanned using ImageScanner III (GE Healthcare, Uppsala, Sweden) and analysis of protein spot volume was performed using ImageMaster Platinum 7.0 software (GE Healthcare, Uppsala, Sweden). Image analysis was restricted to protein spot clusters that appeared consistently within each group of hair shaft proteins. The levels of proteins in each sample were calculated as a percentage of volume contribution (% vol) in which the volume of contribution refers to the volume percentage of a protein taken against the total spot volume of all the proteins, in order to eliminate the possible variations due to staining and/or protein loading. Automatic spot detection was performed with default parameters setting (cut-off parameters were Smooth-2; Saliency-1; Min area-5), whereas spot editing and removal of artefacts were done manually.

Data were analysed using the Statistical Package for Social Sciences (SPSS) version 25.0 (IBM Corporation, New York, USA). Shapiro–Wilk test of normality was used to assess the distribution of the datasets. Parametric Student’s *t*-test and ANOVA was used to analyse and identify significant changes in expression between two population means (gender) and several population groups (ethnicities), respectively, when the normality assumption was met (*p* > 0.05). Following ANOVA, a pairwise comparison procedure was performed to test all possible pairwise differences of the means using either Tukey HSD when equal variances was assumed or, Dunnett’s T3, if otherwise. On the other hand, non-parametric Mann–Whitney U and Kruskal–Wallis tests with rank-based *post-hoc* test was used as their respective counterparts when the assumption for normality was violated (*p* < 0.05). All values were presented as mean  ± 95% confidence interval, unless otherwise stated. A *p* value of less than 0.05 and fold change of more than 1.5-fold was considered significant.

### Mass spectrometry and database search

Identification of proteins was performed as previously described with minor modifications (Seriramalu et al., 2010). Briefly, protein spots of interests were carefully cut out from 2-dimensional electrophoresis gels and kept in high-purity water at −20°C. Gel plugs were first destained using 15 mM potassium ferricyanide (III) and 50 mM sodium thiosulphate for 15 min at room temperature. The destaining procedure was repeated until the gel plugs became clear and transparent. The proteins in gel plugs were then reduced and alkylated using 10 mM DTT and 55 mM iodoacetamide both in 100 mM ammonium bicarbonate. They were then washed thrice with 50% acetonitrile in 100 mM ammonium bicarbonate, dehydrated with 100% acetonitrile and dried using vacuum centrifugation. The dried gels were treated with trypsin (6 µg/mL in 50 mM ammonium bicarbonate) for 18 h at 37 °C. The resulting peptides were then dried, reconstituted in formic acid (0.1%) and desalted using ZipTip with C18 resin (Millipore, Massachusetts, USA). The desalted and concentrated peptides were mixed with equal volume of *α*-cyano-4-hydroxycinnamic acid (6 mg/ml), before being spotted onto the OptiToF 384-well insert (0.7 µl) of the 5800 MALDI ToF/ToF analyser (SCIEX, Framingham, USA).

The proteins were identified using MASCOT search engine (Perkins et al., 1999) and the resulting mass spectral data were thoroughly queried against the human hair entries in the Uniprot database (last update: January 17, 2019, containing 1329 sequences). The following parameters were set: enzyme: trypsin; maximum missed cleavages: 1; fixed modification: carbamidomethylation of cysteine; variable modification oxidation of methionine; precursor ion mass tolerance: 100 ppm; fragment ion mass tolerance: 0.2 Da. An individual ion score of more than 17 indicates extensive homology or identity (*p* < 0.05).

### Verification of altered protein abundance using immunoblotting

Immunoblotting was performed on pooled hair shaft protein samples of each groups of subjects, to verify the altered abundance of proteins observed in the 2-dimensional electrophoresis analysis. A total of 30 µg of proteins for each pooled sample, determined using Bio-Rad protein assay (Bio-Rad, California, USA), was separated on 12.5% SDS polyacrylamide gel and transferred onto PVDF membranes (0.2 µm, PALL Life Sciences, Port-Washington, USA) using a Mini Trans-Blot® Electrophoretic Transfer Cell (Bio-Rad, Hercules, USA) for 1 h at 100 V. Membranes were stained for total protein using MemCode™ Reversible Protein Stain Kit (Thermo Fisher Scientific, Waltham, USA), dried and scanned to be used as a loading control. After removal of Mem-Code™ staining, the membranes were blocked with 5% skim milk in 1 × TBST (25 mM Tris, 500 mM NaCl, 0.05% Tween, pH 7.5) for 30 min, washed thrice, 5 min with 1 × TBST, and incubated with anti-KRT86 antibody (ab192754) (Abcam, Cambridge, UK) overnight at 4 °C. After washing with 1 × TBST thrice for 5 min each wash, the membranes were incubated for 1 h with horseradish peroxidase conjugated goat anti-guinea pig IgG H&L antibody (ab6908) (Abcam, Cambridge, UK). The membranes were washed again thrice, 5 min with 1 × TBST and developed either using 3, 3′-diaminobenzidine chromogen solutions (Thermo Fisher Scientific, Waltham, USA) containing 0.02% hydrogen peroxide or WesternBright Sirius enhanced chemiluminescence system (Advansta, California, USA). MemCode™ signal and protein band intensities were obtained using the ImageJ Software (NIH, Bethesda, USA).

## Results

### Hair shaft protein profiling—distinguishing genders and ethnicities

The extraction yields of protein (%) using the alkaline solubilisation method as previously described by Wong et al. (2016) were generally higher compared to the methods of Lee, Rice & Lee (2006) and Shindai (Fujii & Daguang, 2008). When alkaline solubilisation protein extraction method was applied to 5 mg of hair strands from 40 different individuals (30 females and 10 males), an average extraction yield of 63.56  ± 6.59 (mean  ± SD) was obtained.

**Figure 1:**
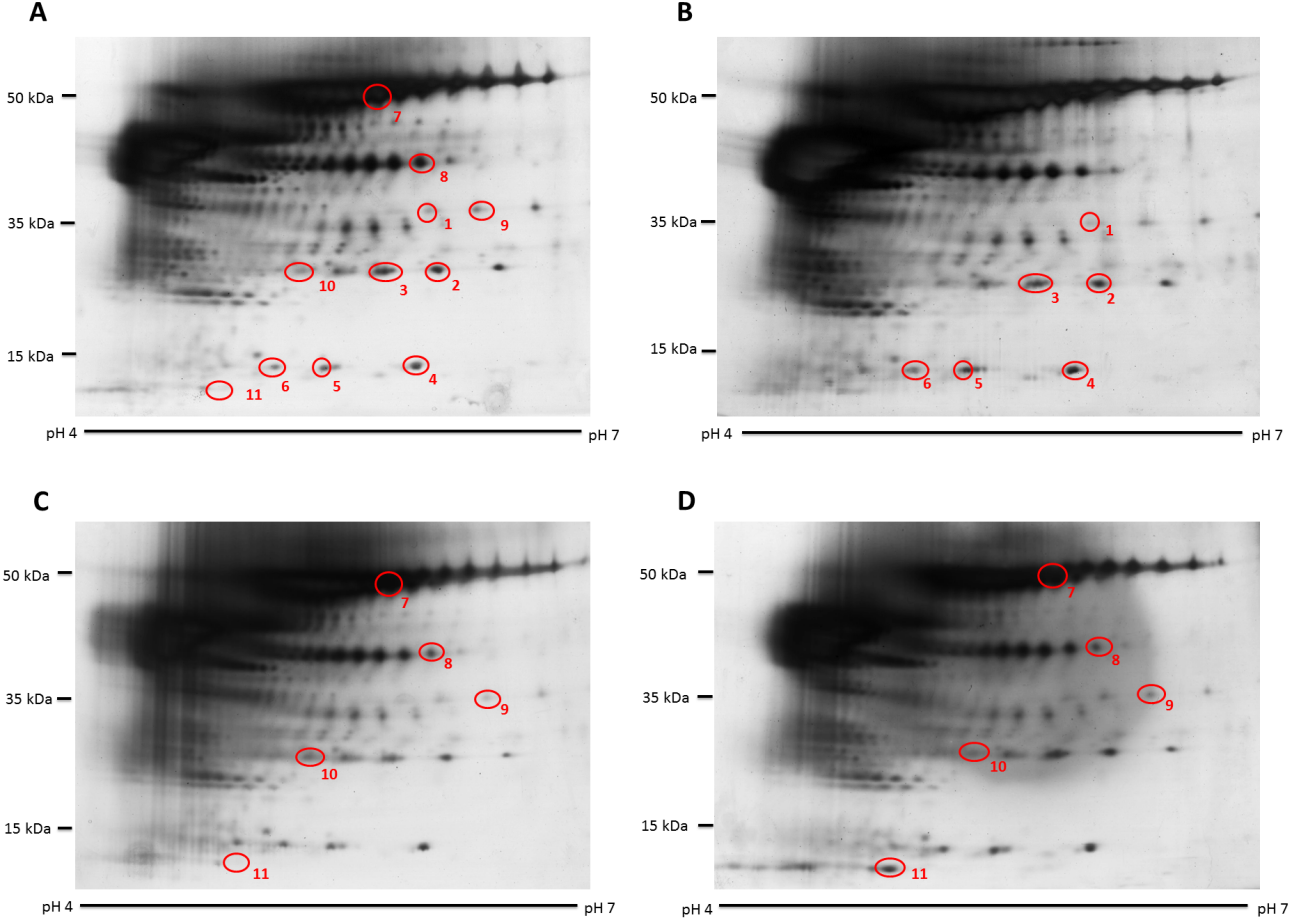
Representative hair shaft protein profiles. (A) Circles indicate 11 protein spots that were significantly different in abundance between gender [male (*n* = 10) and female (*n* = 10) subjects of the same ethnicity (Malaysian Malay)] (spots 1–6) and three different ethnicities [Malaysian Malay (*n* = 10), Chinese (*n* = 10) and Indian (*n* = 10) female subjects] (spots 7–11). (B, C and D) Representative hair shaft protein profiles of male Malay, female Chinese and Indian subjects, respectively.

**Figure 2:**
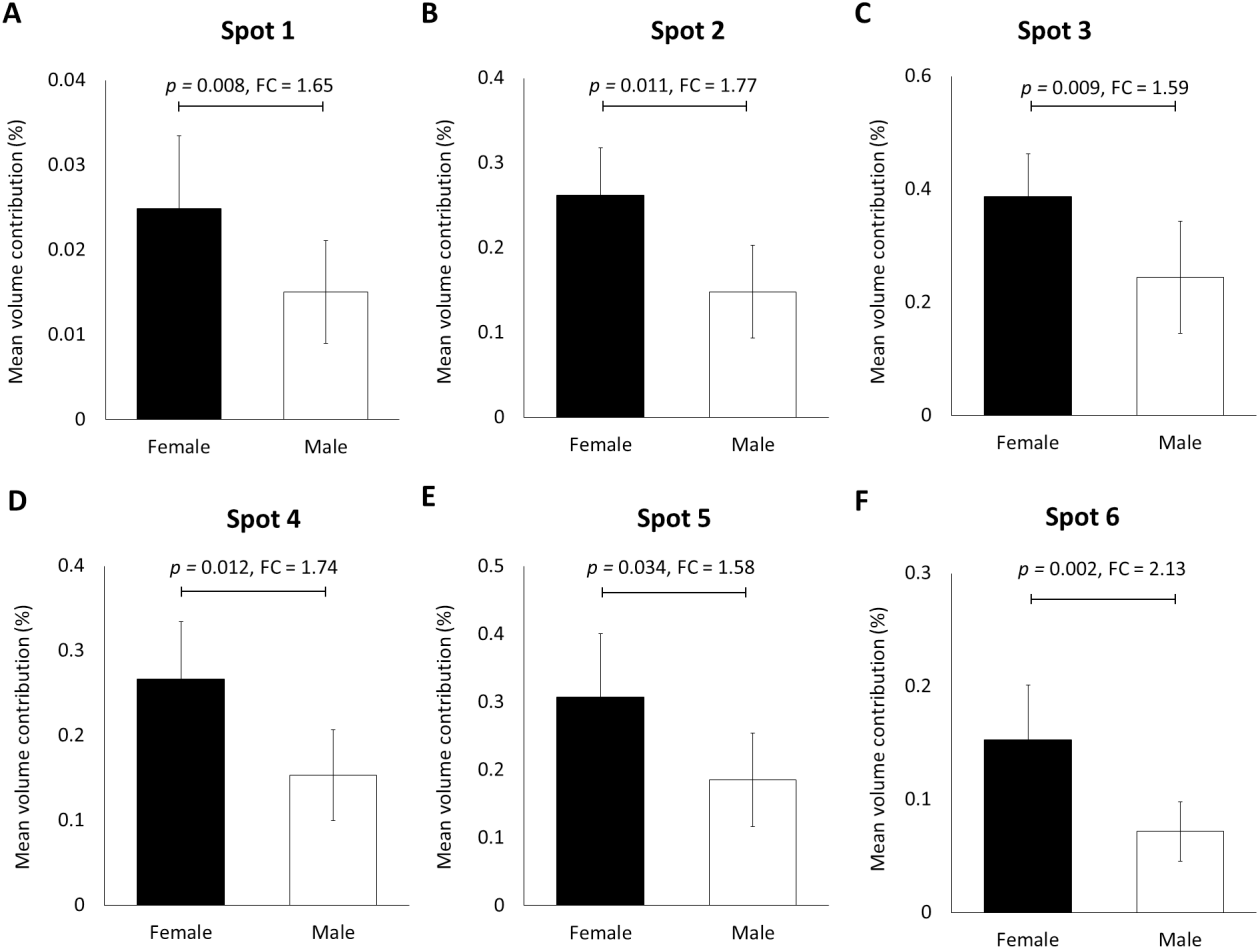
Volume contribution of 6 hair shaft protein spots that were significantly different between male and female subjects (Fig. 1). Gel images were analysed by ImageMaster 2D Platinum Software (mean ± 95% confidence interval; *n* = 20). (A–F) Six protein spots (K85, three protein species of K86 and two protein species of K81) that were significantly different in abundance between male and female subjects. FC is fold change between the mean values for males and females. Error bars represent 95% confidence intervals.

When the human hair shaft protein extracts from female and male subjects of the Malay ethnic group were separated by 2-dimensional electrophoresis and subjected to silver staining, similar profiles were obtained (Figs. 1A and B). The gel profiles were then subjected to ImageMaster 2D Platinum Software and statistical analyses (see section: ‘Data Analysis’). Abundance of 6 hair shaft protein spots was found to be significantly higher in female compared to male subjects (Fig. 2). Spot 3 showed the highest mean percentage of volume contribution for females compared to males. The highest fold change difference was observed in spot 6 (Fig. 2).

**Figure 3:**
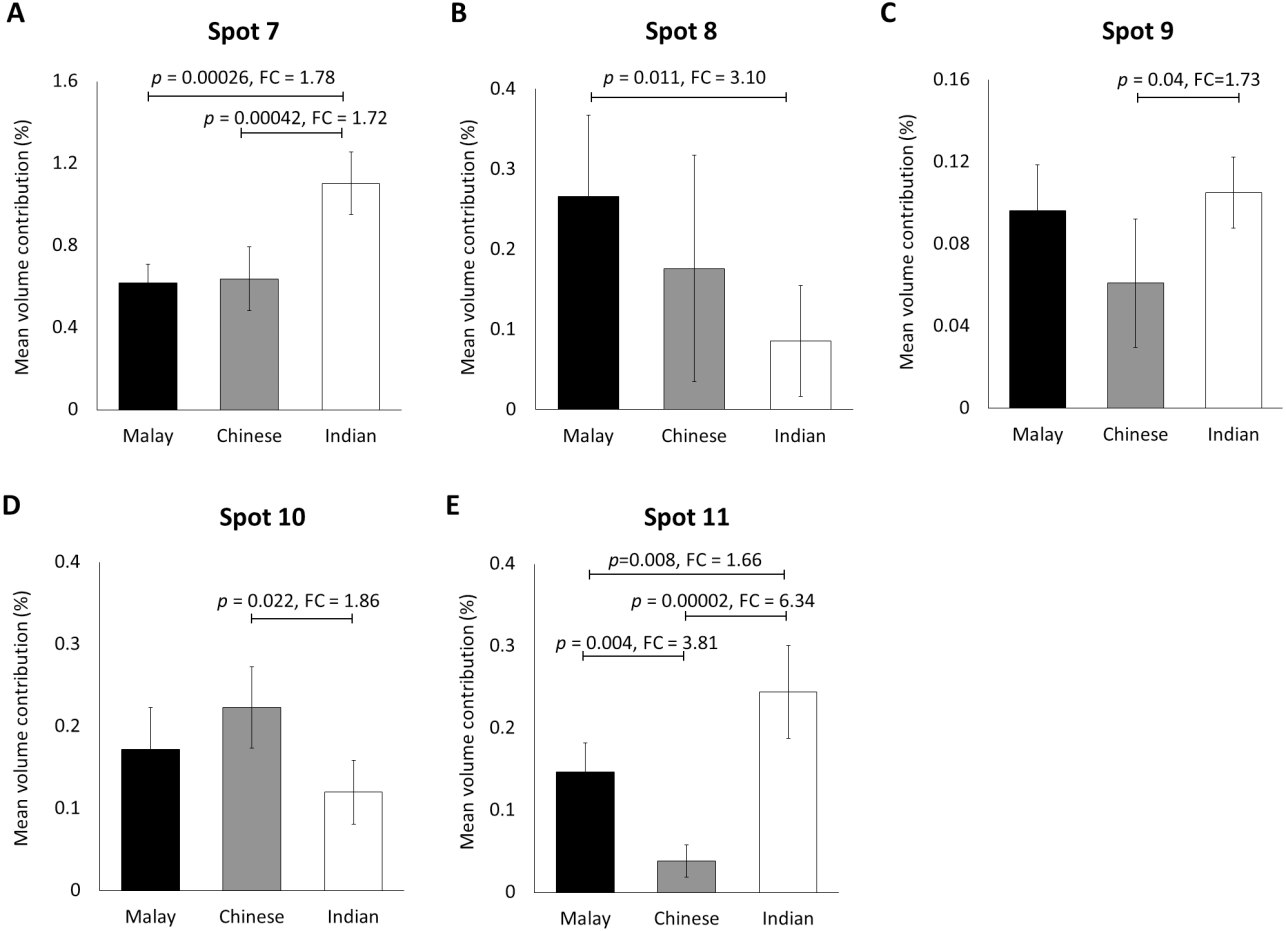
Volume contribution of hair shaft protein spots that were significantly different between the three different ethnicities. The five protein spots (two protein species of K86, K83, K81 and K33B corresponding to the spot numbers 7–11 shown in Figs. 1B–1D, respectively) of significant altered abundance between Malay (*n* = 10), Chinese (*n* = 10) and Indian (*n* = 10) subjects as analysed by ImageMaster 2D Platinum Software of gel profiles (mean ± 95% confidence interval) were demonstrated (A–E). FC is fold change between the mean values. Error bars represent 95% confidence intervals.

When the same gel profiles of the female Malay subjects were compared to those generated from subjects of Chinese and Indian ethnicities of the same age range, 5 protein spots were significantly different in at least one ethnic group compared to the others (Fig. 3). Among the protein spots of altered abundance, spot 7 appeared the most intense. However, spot 11 demonstrated the highest fold change differences between Indian (0.244  ± 0.028) and Chinese (0.038  ± 0.079) subjects, as well as between Malay (0.147  ± 0.042) and Chinese (0.038  ± 0.027) subjects. When taken together, the analysis generally showed that the Indian female subjects had the highest mean percentage of volume contribution for spots 7, 9 and 11 compared to other ethnicities (females) whilst their spots 8 and 10 were the least intense. The Chinese subjects showed the highest mean percentage of volume contribution in spot 10 and lowest values for spots 9 and 11. In the Malay female subjects, spot 8 had the highest mean percentage of volume contribution, while spot 7 was the least intense.

### Identification of hair shaft proteins by MALDI-ToF/ToF

The hair shaft protein spots of significant altered abundance between subjects of different genders and ethnicities were identified using MALDI-ToF/ToF analysis and search of the human hair database (Table 1). Analysis of the 11 hair shaft proteins of interest (Fig. 1) identified five different types of keratins, including (1) type II cuticular Hb6 (K86) for spots 3, 4, 5, 7 and 8, (2) type II cuticular Hb3 (K83) for spot 9, (3) type II cuticular Hb1 (K81) for spots 2, 6 and 10, (4) type II cuticular Hb5 (K85) for spot 1 and (5) type I cuticular Ha3-II (K33b) for spot 11. While the hair shaft of subjects of different genders demonstrated significant altered levels of K81, K85 and K86, those from different ethnicities showed significant different abundance of K33b, K81, K83 and K86.

**Table 1:** MS identification of 2-dimensional electrophoresis hair shaft protein gel spots of altered abundance. Spots that were significantly different in abundance between subjects of distinct genders (spots 1–6) and ethnicities (spots 7–11) were excised from gels and subjected to in-gel trypsin digestion, MALDI-ToF/ToF analysis and human hair database query (Fig. 1). Experimental mass was calculated based on relative mobilities (*R*_*f*_) of the spots. cov, coverage; p*I*, isoelectric point.

**Spot number**	**Accession number**	**Protein name**	**Abbreviation**	**Mascot score**	**Sequence cov (%)**	**Distinct peptides**	**Theoretical mass (Da)**	**Experimental mass (Da)**	**Theoretical p*I***	**Experimental p*I***
1	P78386	Keratin, type-II cuticular Hb5	K85	74	6	4	57,306	32,359	6.28	5.90
2	Q14533	Keratin, type-II cuticular Hb1	K81	51	3	2	56,832	24,547	5.40	6.00
3	O43790	Keratin, type-II cuticular Hb6	K86	106	10	4	55,120	24,547	5.56	5.70
4	O43790	Keratin, type-II cuticular Hb6	K86	105	15	5	55,120	13,803	5.56	5.70
5	O43790	Keratin, type-II cuticular Hb6	K86	49	7	3	55,120	13,803	5.56	5.30
6	Q14533	Keratin, type-II cuticular Hb1	K81	42	1	1	56,832	13,803	5.40	5.00
7	O43790	Keratin, type-II cuticular Hb6	K86	509	35	15	55,120	54,954	5.56	5.50
8	O43790	Keratin, type-II cuticular Hb6	K86	164	11	7	55,120	39,810	5.56	5.90
9	P78385	Keratin, type-II cuticular Hb3	K83	55	2	2	55,928	32,359	5.54	6.30
10	Q14533	Keratin, type-II cuticular Hb1	K81	51	3	2	56,832	24,547	5.40	5.20
11	Q14525	Keratin, type-I cuticular Ha3-II	K33b	18	2	1	47,338	11,749	4.81	4.80

### Verification of K86 abundance

**Figure 4:**
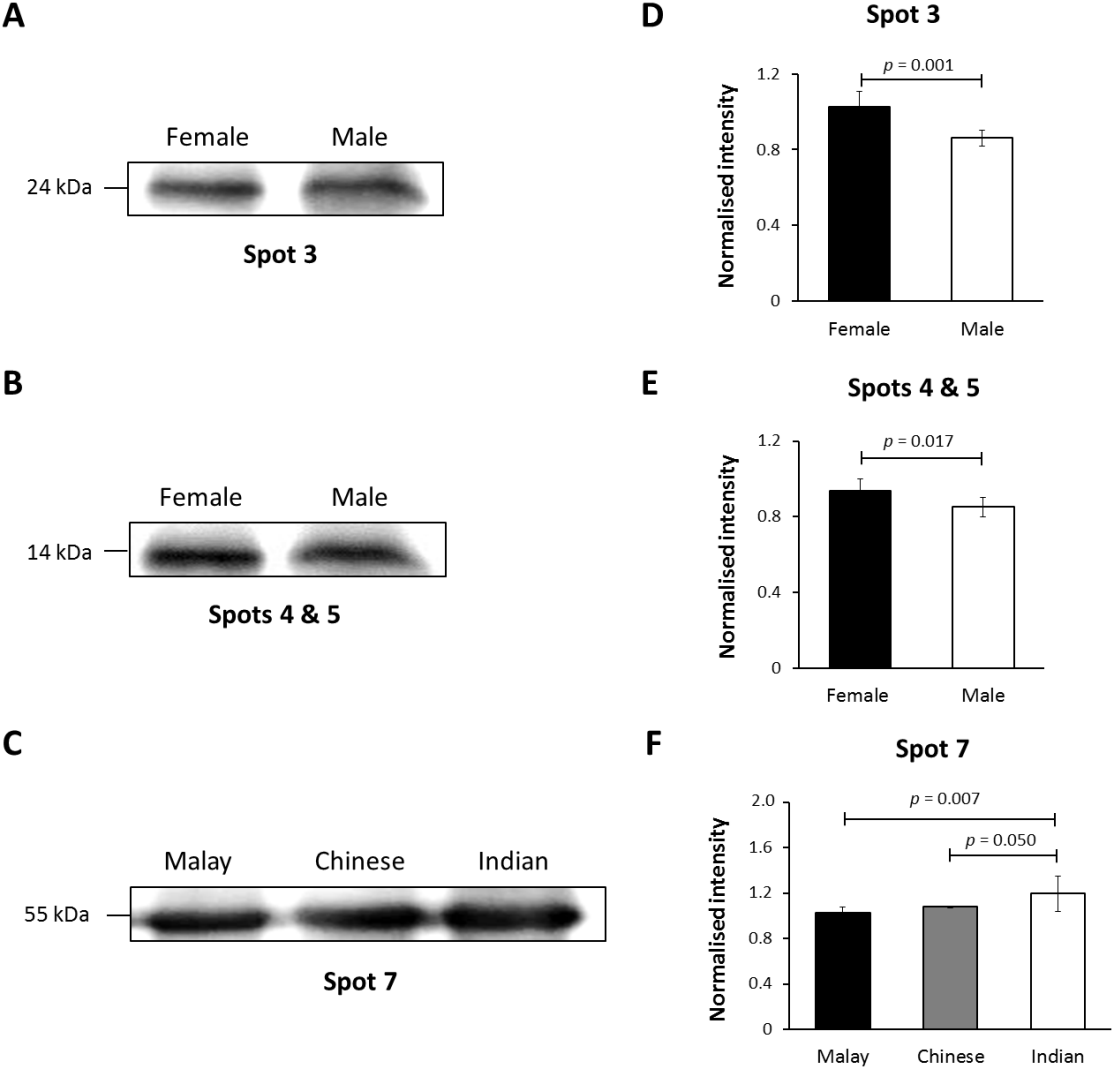
Immunoblotting of hair shaft K86. Pooled hair shaft proteins were initially subjected to SDS-polyacrylamide gel electrophoresis and transferred electrophoretically onto PVDF membranes. Immunodetection was performed using polyclonal guinea pig antisera against K86, whilst peroxidase-conjugated goat anti-guinea pig antibody was used as secondary antisera. The PVDF membranes was developed using WesternBright Sirius enhanced chemiluminescence system. Normalised intensity values are ratios of the relative intensities of the bands to their respective loading controls (intensities of total protein using MemCode™ Reversible Protein Stain Kit). (A, B and C) Cropped images of bands; (D, E & F) normalised intensities of the four K86 peptides (spots 3, 4, 5 and 7, respectively). Error bars represent 95% confidence intervals.

In this study, K86 was selected for verification of its abundances in the hair shaft of the subjects using immunoblotting method in view of its highest altered frequency as well as availability of commercialised antibodies against the protein. However, since the anti-K86 used is known to specifically recognise amino acids 446-469 of the keratin peptide, one of its peptide spots (spot 8), with amino acid sequences not in the region recognised by the antisera (Table S1), was not included in the analysis. Fig. 4 demonstrates the representative images generated from the immunoblotting experiments. The results obtained for K86 spots (3, 4, 5 and 7) appeared to corroborate the findings previously obtained via two-dimensional gel electrophoresis separation. K86 bands equivalent to the molecular weights of spots 3, 4 and 5 (spots 4 and 5 share similar molecular weight) appeared intensely stained in the female Malay subjects, relative to the male counterparts, while the female Indian subjects demonstrated more intense K86 bands compared to the female Malay and Chinese subjects. When analysed by densitometry, the intensity ratios for female Malay:male Malay bands for spots 3-, 4- and 5-equivalent K86 bands were <1.5-fold different, but significant (*p* < 0.05). On the other hand, the intensity ratios for the K86 spot 7-equivalent band for female Malay versus Indian subjects showed >1.5-fold difference (FC = 1.62, *p* = 0.008). The levels of spot 7-equivalent bands seemed comparable between the female Malay and Chinese (*p* > 0.05) subjects.

## Discussion

Protein profiling of the human hair shaft have shown promising avenue for providing definitive identification in aid of forensic investigations (Wu et al., 2017). Formed via cornification of keratinocytes, the hair shaft mainly contains 17 different types of keratins, (i.e., K31-40 (including K33a and K33b) and K81-86) (Moll, Divo & Langbein, 2008), which are poorly analysed mainly because of their limited solubility and extraction yield. In the present study, we have adopted a newly developed protocol that enhanced the extraction yield of proteins from the human hair shaft (Wong et al., 2016) and reanalysed the material. The gel profiles that were generated showed reasonably improved resolution of the separated hair shaft proteins compared to those that were earlier reported (Barthélemy et al., 2012; Thibaut et al., 2009; Nakamura et al., 2002). Identification of these proteins by mass spectrometry and search of the human hair protein database showed that they comprised the different types of keratins, the cysteine-rich helicoidal proteins that protect the hair because of their insolubility and impermeability.

In view of its reproducibility, the profiling of the human hair shaft proteins was further utilised in a pilot study to determine if the developed profiles could be used to distinguish gender of individuals. Our analysis of 20 hair shaft samples from healthy individuals between the age of 21–40 years demonstrated significant higher abundance of six different type-II keratin spots, including two K81, one K85 and three K86, in the women subjects compared to men. These different types of keratins are known to be restricted to the hair shaft and not present in the follicle (Moll, Divo & Langbein, 2008). However, all these spots of altered abundance appeared to be truncated or processed keratin products as they were resolved within the molecular weight regions lower than their putative primary translated precursor polypeptides (Table 1). The influence or effects of processing of proteins in this material remains to be elucidated in future investigations.

Similar marked shifts in molecular weights and isoelectric point (p*I*) values of the hair shaft proteins that were separated by 2-dimensional electrophoresis, which may be due to deamidation, post-translational modifications, or processing at the RNA level as sequence variants (Person et al., 2006), have been previously reported (Barthélemy et al., 2012). Whether this was an effect of the strong alkali used at 90 °C in the hair shaft protein extraction, or that the proteins were further biologically processed during their various stages of genetic expression, subsequent to their translation or during cornification, remains to be investigated.

The gel electrophoresis profiling of 30 hair shaft samples taken from women of similar age range but from three distinctive Malaysian ethnic subpopulations further showed significant altered abundance of one type-I (K33b) and four type-II (K81, K83 and two K86) keratins between the ethnic groups that were analysed. Like the earlier detected keratins, the type-I K33b and all the type-II keratins detected are also known to be localised exclusively in the hair shaft (Moll, Divo & Langbein, 2008). Based on the resolved experimental molecular weights, all the five spots of altered abundance also appeared to be truncated or processed keratins. In addition, the K81 and K86 spots that were also detected in this analysis were different from their counterparts that were detected in the earlier gender analysis as they showed distinctive experimental molecular weight and p*I* values.

The results of the latter study also demonstrated that the Indian ethnic group to be most distinctive as they showed four abundantly different keratins (K33b, K81, K83 and K86 (spot 7)) compared to the Chinese and three keratins that were differently altered (K33b and two K86 (spots 7 and 8)) compared to the Malays. On the other hand, the Chinese and Malay ethnic groups only appeared to be distinctive in the abundance of K33b (3.81-fold of difference) and their levels of the type-I keratin were both significantly different compared to the Indians. These results are generally comparable with the genetic data that were earlier reported. In a study using multi-dimensional scale analysis on the population genetic structure of the different ethnic groups in Peninsular Malaysia, Hatin et al. (2011) had previously reported that the Malay and Chinese populations were clustered together while the Indians were further apart.

Data generated from the verification study using immunoblotting method generally substantiated the abundances of the K86 truncated peptides in the hair shaft of subjects previously obtained via two dimensional gel separation method, with little variation in the levels of significance and/or intensity ratios (FC). The intensity of spots 3-, 4-, 5- and 7-equivalent K86 peptide bands appeared to corroborate the findings previously obtained via two-dimensional gel electrophoresis separation. Together, these data further emphasise the potential of the keratin peptide markers for distinguishing gender and ethnicity, and show promises for future forensic applications.

## Conclusion

When taken together, the human hair keratin profiling that was conducted in this pilot study provided a potential method that can be used to distinguish gender and ethnicity of individuals based on their hair shaft samples. However, a larger scale analysis of the hair shaft proteins using antibodies that are specific to the different types of keratins that were highlighted in the present study would be needed to increase the robustness of the results. This large scale study could eventually lead to the development of a searchable database as well as signature keratin biomarkers that could facilitate determination of one’s gender and ethnicity based on their hair shaft keratin profiles. Nonetheless, several other factors such as the effects of chemical exposure as well as dietary and environmental influences on the hair shaft keratin profiles are also required for confirmation of the accuracy of the results.

##  Supplemental Information

10.7717/peerj.8248/supp-1 Data S1 Results of analysis of protein spot volume Analyses were performed using ImageMaster Platinum 7.0 software.

10.7717/peerj.8248/supp-2 Figure S1A Uncropped 2DE gel image of a female Malay subject

10.7717/peerj.8248/supp-3 Figure S1B Uncropped 2DE gel image of a male Malay subject

10.7717/peerj.8248/supp-4 Figure S1C Uncropped 2DE gel image of a female Chinese subject

10.7717/peerj.8248/supp-5 Figure S1D Uncropped 2DE gel image of a female Indian subject

10.7717/peerj.8248/supp-6 Figure S2A Uncropped image of a PVDF membrane stained for total protein

10.7717/peerj.8248/supp-7 Figure S2B Uncropped image of a PVDF membrane developed using WesternBright Sirius enhanced chemiluminescence system

10.7717/peerj.8248/supp-8 Table S1 ToF/ToF derived peptide sequences of 36 kDa K86 protein (spot 8)

## Additional Information and Declarations

### Data Availability

The following information was supplied regarding data availability: The raw data is available in the Supplemental Files.
