# Supplementary figures and images for: Potential use of human hair shaft keratin peptide signatures to distinguish gender and ethnicity
Source: PeerJ. 2020 Jan 30;8:e8248. doi: 10.7717/peerj.8248 (PMC6995659; doi:10.7717/peerj.8248)

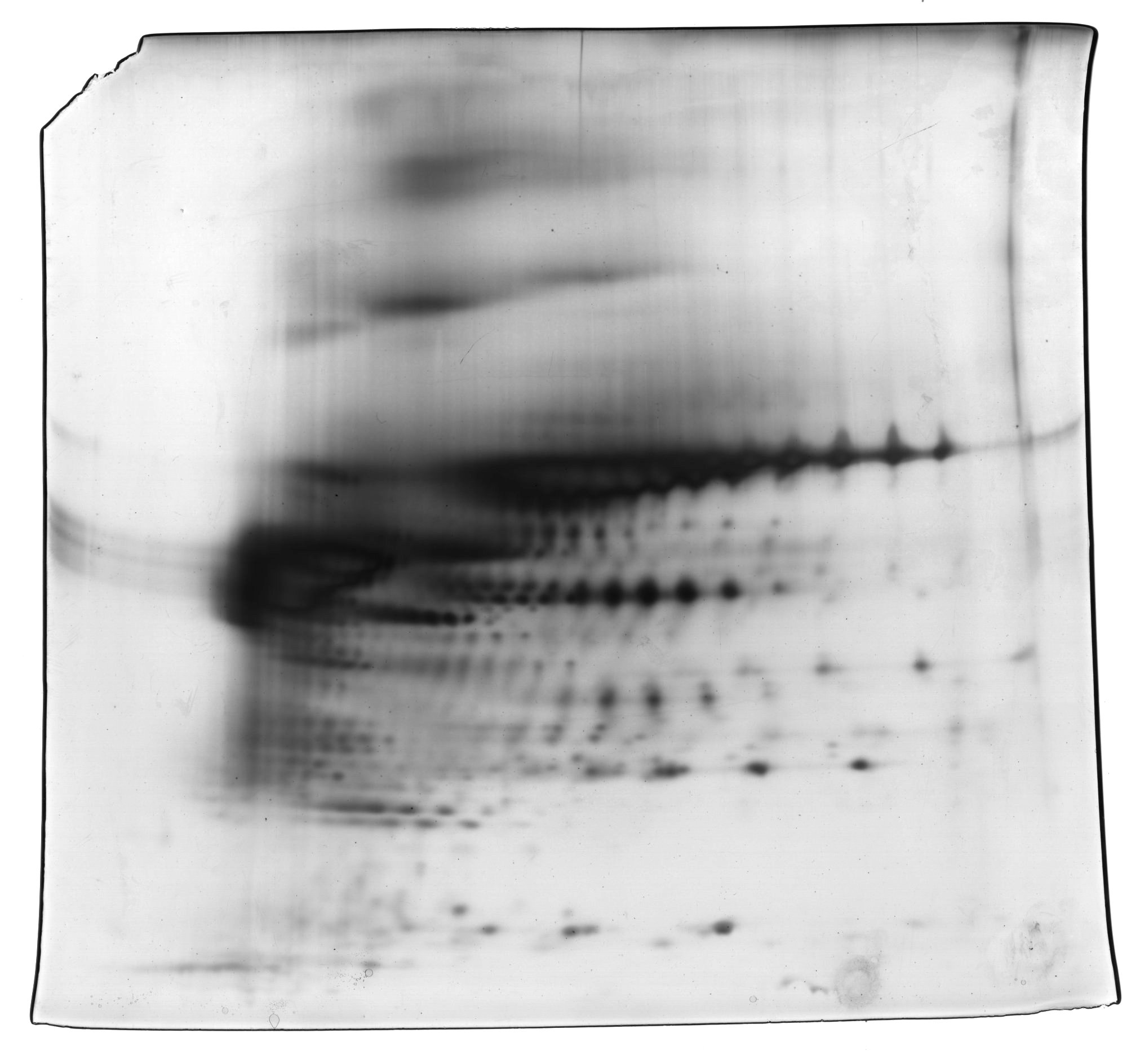

Supplement: Figure S1A [file peerj-08-8248-s002.jpg]

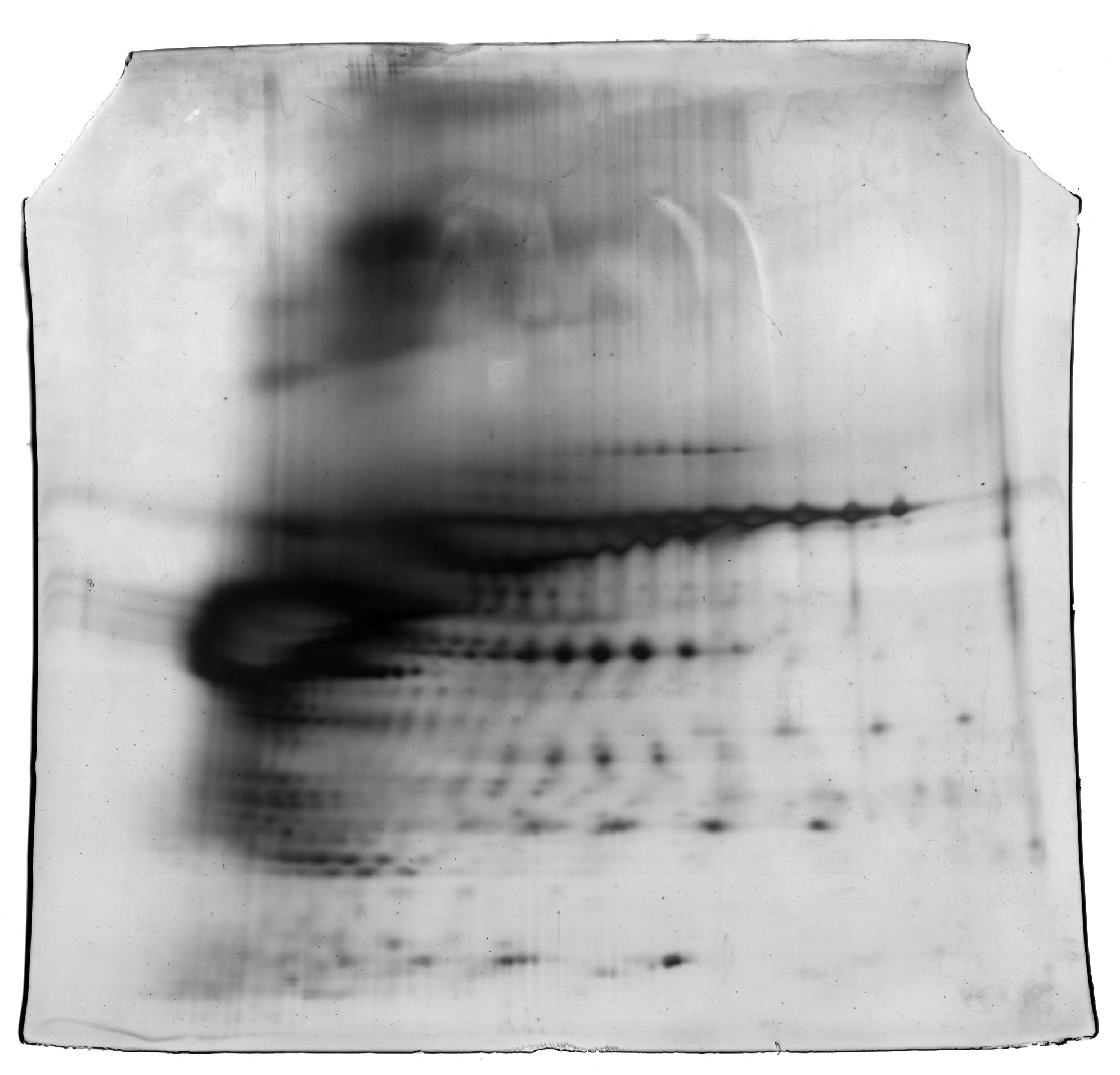

Supplement: Figure S1B [file peerj-08-8248-s003.jpg]

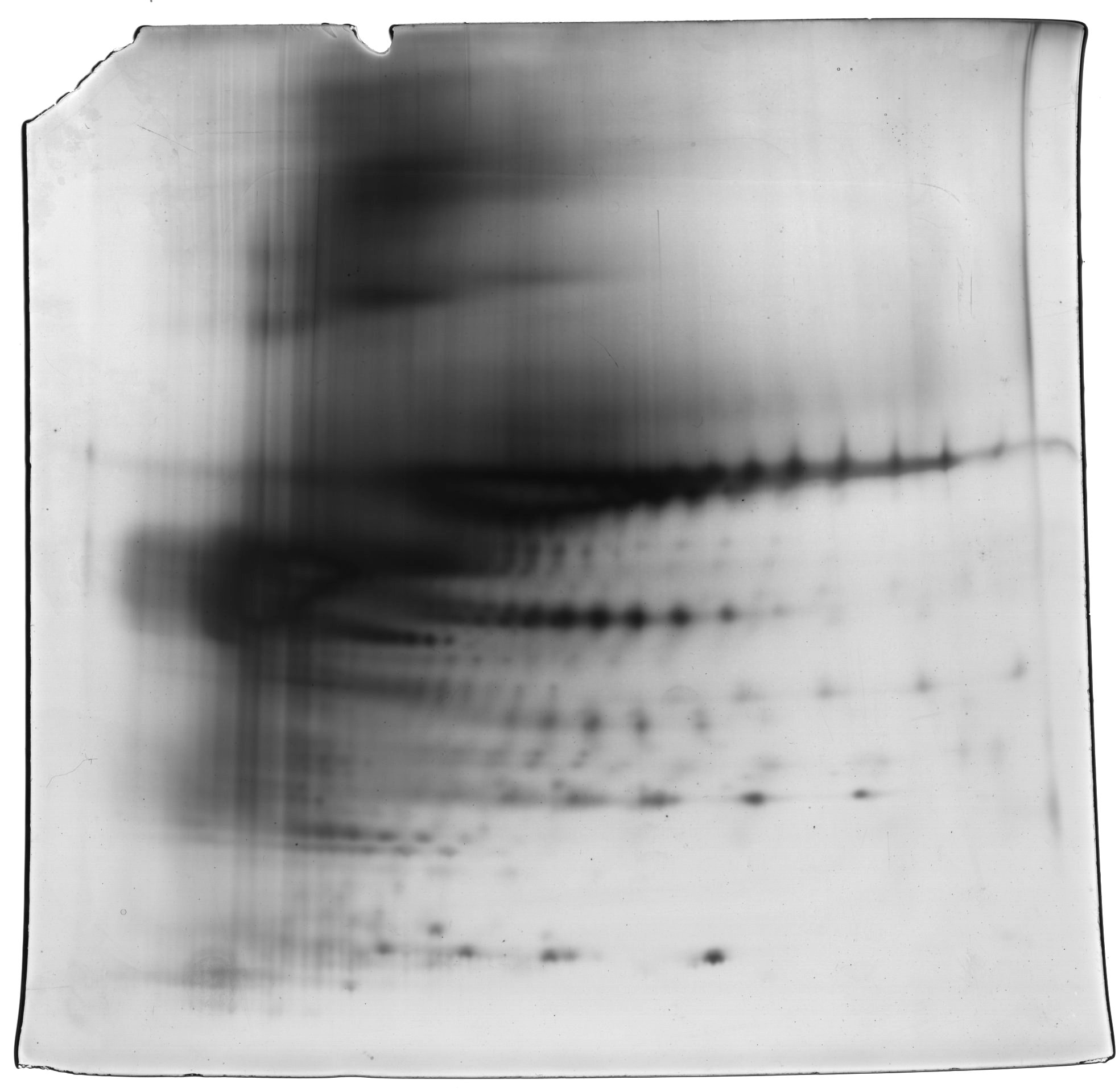

Supplement: Figure S1C [file peerj-08-8248-s004.jpg]

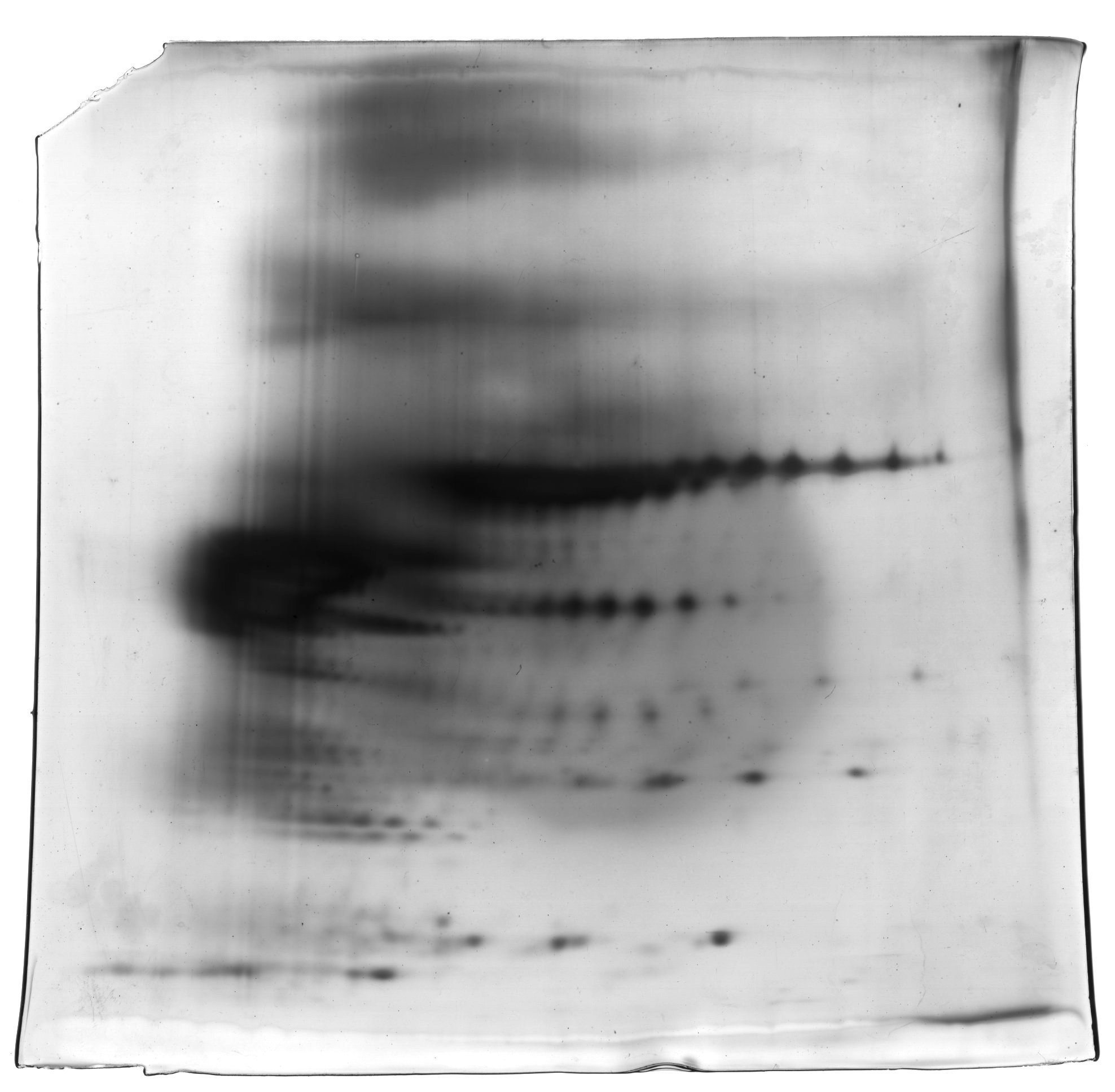

Supplement: Figure S1D [file peerj-08-8248-s005.jpg]

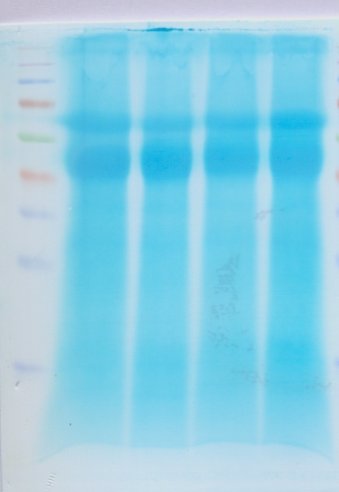

Supplement: Figure S2A [file peerj-08-8248-s006.jpg]

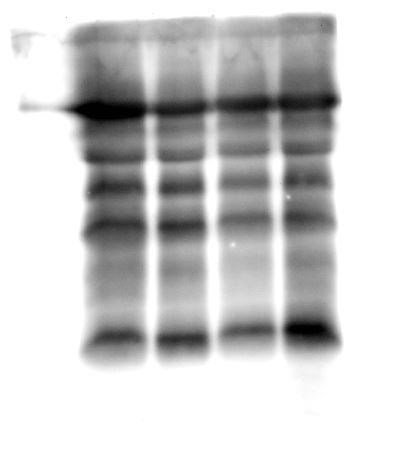

Supplement: Figure S2B [file peerj-08-8248-s007.jpg]
