# Supplementary material for: Potential use of human hair shaft keratin peptide signatures to distinguish gender and ethnicity
Source: PeerJ. 2020 Jan 30;8:e8248. doi: 10.7717/peerj.8248 (PMC6995659; doi:10.7717/peerj.8248)
Supplement: Table S1 [file peerj-08-8248-s008.docx]

**Supplemental Table 1. ToF/ToF derived peptide sequences of ~36 kDa K86 protein (spot 8).**

| **Peptide sequences** | **Amino acid** |
| --- | --- |
| **R.CCITAAPYR.G** | 25-33 |
| **R.FLEQQNK.L** | 126-132 |
| **K.LQFYQNR.E** | 138-144 |
| **R.EAECVEADSGR.L** | 165-175 |
| **R.LYEEEIR.V** | 242-248 |
| **K.AQYDDIVTR.S** | 280-288 |
| **R.AEAESWYR.S** | 291-298 |
|  |  |
